# Opioid Safety and Concomitant Benzodiazepine Use in End-Stage Renal Disease Patients

**DOI:** 10.1155/2019/3865924

**Published:** 2019-10-20

**Authors:** Rupam Ruchi, Shahab Bozorgmehri, Tezcan Ozrazgat-Baslanti, Mark S Segal, Ashutosh M Shukla, Rajesh Mohandas, Sanjeev Kumar

**Affiliations:** ^1^Division of Nephrology, Department of Medicine, University of Florida, Gainesville, FL, USA; ^2^Division of Nephrology, Department of Medicine, NF/SG Veteran Healthcare System, University of Florida, Gainesville, FL, USA; ^3^Division of Pain Medicine, Department of Anesthesiology, University of Florida, Gainesville, FL, USA

## Abstract

**Background:**

Opioid use is common in end-stage renal disease (ESRD) patients. However, safety of individual opioids and concomitant benzodiazepine use has not been studied.

**Objective:**

To study the epidemiology of opioid and concomitant benzodiazepine use in ESRD population. To study the clinical safety profile of individual opioids in patients on hemodialysis.

**Design:**

Retrospective analysis of the U.S. Renal Data System. A comprehensive review of the current literature was performed to update currently used opioid safety classification.

**Participants:**

ESRD patients ≥18 years on hemodialysis who were enrolled in Medicare A and B and Part D between 2006 and 2012, excluding those with malignancy.

**Main Measures:**

Hospital admission with diagnosis of prescription opioid overdose within 30, 60, and 90 days of prescription; death due to opioid overdose.

**Results:**

Annually, the percentage of patients prescribed any opioid was 52.2%. Overall trend has been increasing except for a small dip in 2011, despite which the admissions due to opioid overdose have been rising. 30% of those who got a prescription for opioids also got a benzodiazepine prescription. 56.5% of these patients received both prescriptions within a week of each other. Benzodiazepine use increased the odds of being on opioids by 3.27 (CI 3.21–3.32) and increased the odds of hospitalization by 50%. Opioids considered safe such as fentanyl and methadone were associated with 3 and 6 folds higher odds of hospitalization within 30 days of prescription. Hydrocodone had the lowest odds ratio (1.9, CI 1.8–2.0).

**Conclusions:**

Concurrent benzodiazepine use is common and associated with higher risk of hospitalization due to opioid overdose. Possible opioid-associated hospital admission rate is 4-5 times bigger in ESRD population than general population. Current safety classification of opioids in these patients is misleading, and even drugs considered safe based on pharmacokinetic data are associated with moderate to very high risk of hospitalization. We propose a risk-stratified classification of opioids and suggest starting to use them in all ESRD patients.

## 1. Introduction

Pain is a distressing symptom in patients with end-stage renal disease (ESRD), affecting over 50% of population [1–4]. It has a negative impact on multiple patient outcomes such as quality of life, depression, and adherence to dialysis [5, 6] that are strongly associated with higher morbidity and mortality [7].

Management of pain in ESRD population is difficult and suboptimal. The modified WHO ladder for pain management in ESRD proposes opioids for moderate-to-severe pain, defined as rating of 5 and above on the visual analog scale [8]. These protocols have been shown to have excellent efficacy for analgesia in about 96% of ESRD patients over 4 weeks but with real potential for opioid misuse and diversion of the prescription, which can further lead to overdose and related complications. Thus, a dichotomous reality exists for the management of pain among patients with ESRD. On one hand, pain is under-managed in this population [9], while on the other hand, injudicious use of opioids either alone or in combination with additional narcotics results in accumulation of the opioid and/or their active metabolites resulting in higher adverse effects and mortality.

The 2012 American Academy of Family Physicians guidelines for opioid use [10] categorically classifies opioids as “safe,” “unsafe,” or “to be used with caution.” However, these guidelines are based on older reports [11], and there is a lack of guidance for many of the modern opioid analgesics. Further, the categorical classification is largely derived from small pharmacokinetic studies done on dialysis patients or case reports, and it lacks the safety demonstrations for their long-term clinical use. Finally, these guidelines do not provide any guidance regarding the concomitant use of benzodiazepines, which could be a marker for substance use, and known risk factors for increased hospitalizations and mortality in general population. However, benzodiazepines could also have legitimate medical use for appropriate symptom control in a chronically ill patient population, and not every prescription should be viewed in the context of a substance use problem in ESRD patients. Little is known about the concurrent opioids and benzodiazepine use and safety in ESRD population.

The overall aim of this study is to characterize the use of opioids alone and in combination with benzodiazepines in ESRD population and to correlate it with their safety outcomes to further assist determination of the pragmatic safety of these drugs in clinical practice.

## 2. Materials and Methods

### 2.1. Study Design, Date Sources, and Patient Selection

**Figure 1:**
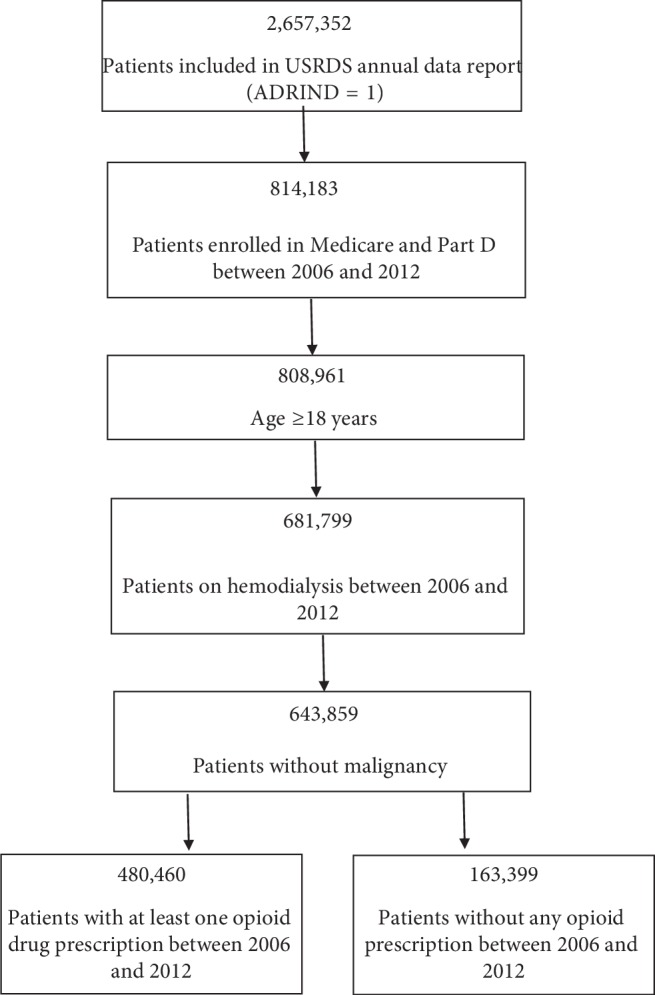
A schematic diagram of patient selection.

We conducted a retrospective study of U.S. Renal Data System (USRDS) database (2014) using the standard analysis files. Using USRDS core dataset, we included ESRD patients with complete identifiers and consistent dates (i.e., annual data reports, ADR = 1). We used treatment history dataset to identify and include ESRD patients ≥18 years of age on hemodialysis who were enrolled in Medicare with Part D coverage between 2006 and 2012. We used ESRD Medicare prescription drug enrollment dataset to obtain information on whether patients were enrolled in Part D in each corresponding year. The demographics and etiology of kidney disease were obtained using core dataset and data on comorbid conditions including diabetes, hypertension, peripheral vascular disease, stroke, and coronary artery disease, and history of smoking, alcohol, and substance abuse was obtained using Centers for Medicare and Medicaid Services (CMS) Medical Evidence Report (CMS-2728). We excluded patients on peritoneal dialysis (PD) and patients with malignancy. The selection of patients is presented in Figure 1.

### 2.2. Opioid Prescriptions

We used ESRD Medicare Prescription Drug Events dataset to obtain information on prescription opioids or benzodiazepines. Only oral and transdermal formulations (e.g., fentanyl patch) of opioids were considered. We considered patients with opioid prescription use if they had at least one opioid prescription throughout the study period. We also examined if a patient had at least an opioid prescription in each of the corresponding year that they were enrolled in Medicare Part D.

### 2.3. Opioid Classification

The available classification of opioids released in 2012 by the American Academy of Family Physicians [10] for ESRD population classifies them as those that are “safe to use” or “unsafe to use.” Opioids with limited data for safety profile are classified as “use with caution.” This classification was based on older data based on pharmacokinetics [11]. We have used this classification as baseline; however, we have modified this to incorporate the newer safety evidence for the existing opioids and to add the newer medications. In order to do this in the most evidence-based possible way, we formed a 2-member expert committee (Kumar specializing in pain medicine and Ruchi specializing in nephrology), both of whom independently reviewed the available literature to update the classification. A detailed updated classification and methodology is referenced in the supplement [12–36] ([Supplementary-material supplementary-material-1]).

### 2.4. Adverse Events

We used hospitalization dataset to identify hospital admission with a diagnosis of prescription opioid overdose using the International Classification of Diseases, Ninth Revision, Clinical Modification (ICD-9 CM) codes. We used the following ICD-9 CM codes: 965.00, 965.02, 965.09, E850.1, and E850.2 for prescription opioid overdose. We also looked at hospital admissions due to prescription opioid overdose within 30, 60, and 90 days of an opioid prescription. The limitation of this method was that the codes for opioid overdose did not indicate if the overdose happened due to intentional self-harm, accidental overdose, or due to deteriorating renal function without adjustment of opioid dosage (there could be several reasons for opioid overdose, which cannot be ascertained by this methodology). We then compared this data with the pharmacokinetic safety class of the individual opioids to see if they corroborate. For this purpose, we categorized the drugs as those associated with mild (OR 1.01–1.9), moderate (OR 2–2.9), high (OR 3–3.9), and very high (OR >4) odds of hospitalization due to opioid overdose. Drugs with OR <1 were considered safe.

For the purpose of this study, we defined death due to opioid overdose as any death due to drug overdose that occurred during a hospitalization with a diagnosis of prescription opioid overdose. We calculated the total length of stay and ICU length of stay using hospitalization dataset. We calculated annual Medicare cost per patient using institutional claims dataset.

### 2.5. Statistical Analysis

Baseline demographics and clinical characteristics were compared between patients with and without opioid prescriptions using one-way analysis of variance (ANOVA) and chi-square tests for continuous and categorical variables, respectively. The annual trend in the proportions of patients with at least one opioid prescription was examined using Cochran–Armitage test for trend. The difference between frequencies and distributions of adverse events due to opioid prescriptions between patients with and without opioid prescriptions were examined using Wilcoxon–Mann–Whitney test for nonnormally distributed continuous variables and chi-square test for categorical variables. The association between patient factors and having at least one opioid prescription were examined using univariate and multivariable logistic regression models. The association between hospital admission with a diagnosis of opioid overdose and odds of opioid prescription drugs within 30, 60, and 90 days was analyzed using univariate and multivariable logistic regression models, which included all opioids and benzodiazepine use odds ratio (OR) and 95% confidence interval (CI), and *p* values were reported. All significance tests were two-sided, with a *p* value < 0.05 considered statistically significant. Statistical analyses were performed using Statistical Analysis Software (SAS) version 9.4 (SAS Institute Inc., Cary, North Carolina).

## 3. Results

### 3.1. Study Cohort and Patient Characteristics

Out of 643,859 patients included in the final cohort, 480,460 (74.6%) patients received at least one prescription of opioid throughout the study period (Figure 1). A total of 6,665,906 opioid prescriptions were written with an average of 13.8 prescriptions per patient. When categorized by annual prescriptions, 52.2% of all patients received at least one opioid prescription. In all, opioids comprised 5.6% of all prescriptions written for these patients. The clinical characteristics of patients with and without opioid prescriptions are presented in Table 1. The association between clinical characteristics and having an opioid prescription is presented in Table 2.

### 3.2. Trends in Opioid Prescription from 2006 to 2012

**Figure 2:**
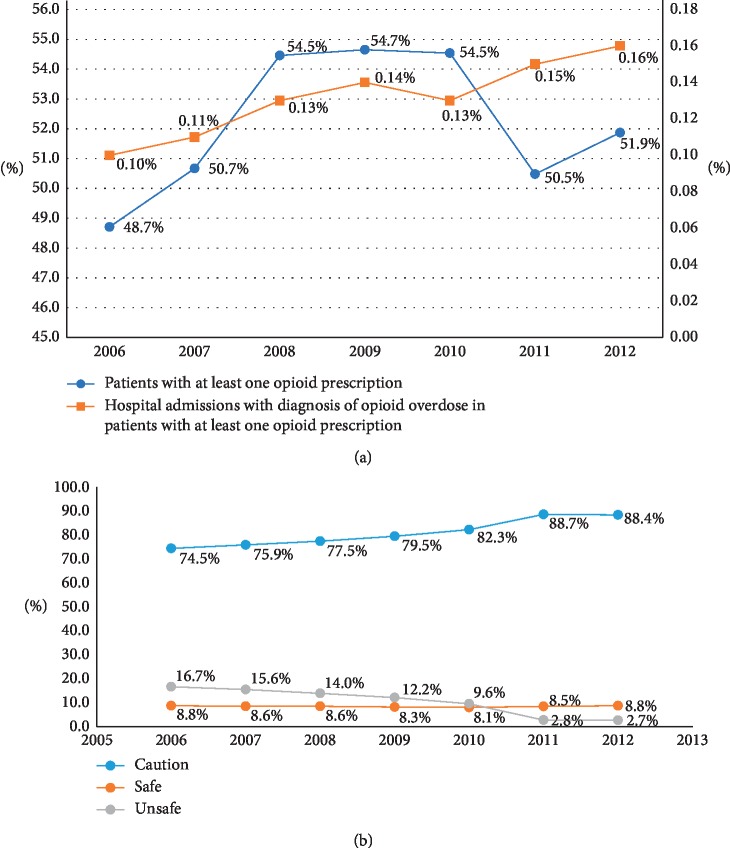
Figures describing the trend of opioid prescription use (a) and class of prescribed opioid from 2006–2012 (b).

Opioid-prescribing rate (OPR), described by CDC as number of prescriptions per 100 persons per year, was 306 in 2012. Over the study period, the OPR increased from 267 in 2006 to 306 in 2012 (*p* < 0.001).  Figure 2(a) shows a significantly rising trend (*p* < 0.001) towards progressively increasing frequency of opioid use both in terms of number of patients getting opioids as well as the total number of opioid prescriptions written.

### 3.3. Safety of Prescribing Patterns

Over the study period, only 8.52% of the opioids prescribed were in the “safe” category, whereas 81.6% of these were in the “use with caution” category and 9.88% were in the “unsafe” category. Overall, there was a trend for decreased use of “unsafe opioids” and greater use of “use with caution” opioids during the study period (*p* < 0.001) (Figure 2(b)).

### 3.4. Adverse Events in Patients Who Received Opioid Prescriptions

Among patients with at least one opioid prescription, 3,231 patients had 4,014 hospital admissions with opioid overdose, with 5 deaths. The median length of hospital and ICU stay due to opioid overdose was 4 (IQR: 2–8) and 2 (IQR 1–4) days, respectively. The annual Medicare cost per patient in this group was $46,000 as compared to $33,000 in those without opioid prescription, *p* < 0.001 (Table 3). In 2006, 10/10,000 hospital admissions were attributed to opioid overdose. This number increased to 13, 15, and 16 per 10,000 hospitalizations in 2010, 2011, and 2012, respectively (*p* trend < 0.001), Figure 2(a).

### 3.5. Association between Opioid Class and Hospitalization from Opioid Overdose

The adjusted odds ratio for hospitalization with prescription opioid overdose within 30, 60, and 90 days of prescription is shown in Table 4. Based on the OR at 30 days, opioids were reclassified into low, moderate, high, and very high risk drugs.

Among the safe category of drugs, fentanyl and hydromorphone were associated with 2.5–3 times higher odds of hospitalization. Methadone was found to be associated with a very high risk of hospitalization at 30 days (OR 5.9) that persisted at 60 and 90 days (OR 5.3 and 4.7, respectively).

Among the unsafe drugs, morphine and meperidine were associated with very high risk of hospitalization. The odds ratio of propoxyphene, tapentadol, and codeine was not significant likely because they accounted for only 0.11%, 0.06%, and 0.02% of all prescriptions ([Supplementary-material supplementary-material-1]).

Among the drugs to be used with caution, hydrocodone had the lowest odds ratio for admission. Oxycodone demonstrated a high risk of hospitalization due to opioid overdose, similar to fentanyl; oxymorphone also resulted in a very high risk of hospitalization. Buprenorphine was associated with a very high risk of hospitalization within 30 days (OR 5.4) that persisted at 90 days.

### 3.6. Concomitant Prescription of Opioid and Benzodiazepines

The largest difference between the two groups (opioid use and nonuse) was found to exist in the use of benzodiazepine, which occurred at 30% in the opioid population compared to 11% in the nonopioid population (*p* < 0.001). 56.5% of individuals who received both a benzodiazepine and opioid prescription, received them within a week of each other. Patients in the opioid group were three times more likely to be on benzodiazepines (adjusted OR 3.271, 95% CI: 3.216–3.326) (Table 2).

### 3.7. Association between Benzodiazepine Prescription and Hospitalization from Opioid Overdose

Having a prescription of benzodiazepines significantly increased the odds of hospitalization due to opioid overdose by 50% within 30, 60, and 90 days (*p* value for all < 0.05). Benzodiazepine use prior to hospitalization was significantly higher in patients who were admitted for opioid overdose compared to those who were admitted without this diagnosis (40% vs. 24%, respectively, *p* < 0.001; OR 2.37; CI 2.21–2.54). There was no significant difference in mortality.

## 4. Discussion

The CDC data on the general population demonstrates that, in 2016, 19.1% of adults received a prescription for opioid, with an OPR of 66 [37]. Based on these statistics, opioid (ab)use is considered a major public health problem. The data of opioid use in ESRD population at the same time is unclear, despite an overall higher prevalence of pain syndromes. In an earlier study of randomly selected patients in 142 DOPPS participating US dialysis units, the fraction of ESRD population prescribed opioids was found to be decreasing, from about 18% in 1997 to 14.9% in 2000, but data on safety were not reported [9]. In one of the recently published USRDS analysis, Kimmel et al. [38] demonstrated that approximately 60% of prevalent ESRD patients received at least one prescription for opioids from 2006 to 2010, suggesting that the opioid problem is at least 4-5 folds higher in the ESRD population as compared to general population.

Considering this burden, our analysis has several unique findings important in highlighting the potential of opioid misuse/overuse in the ESRD population. First, we provide a detailed analysis of the patient characteristics associated with opioid overuse. Specifically, we found that the benzodiazepine use (OR 3.2) has one of the strongest associations with opioid use, whereas those with history of drug dependence (OR 1.3) or smoking (OR 1.3) also suffer from significantly higher opioid use. We additionally found that other conventional socio-medical variables, e.g., race (African Americans), presence of diabetes, peripheral vascular disease or heart disease, and so on, had significant but weaker association (OR 1.03–1.07) with opioid use. This suggests that although pain is a major problem in our ESRD population, the factors that associate strongly with opioid prescription is not their comorbidities and painful conditions, but history of drug dependence, smoking, and benzodiazepine use. These findings corroborate a single center data of 192 patients where the authors reported nearly 52% of patients on opioid having no documented medical indication for their chronic use [39].

Second, our study looks at hospitalizations specifically due to prescription opioid overdose, something that has not been studied before. While Kimmel et al. showed that opioid use is associated with higher overall hospitalization and mortality, our study shows that it increases hospitalization due to prescription opioid overdose, and the odds of this increases by 50%, with concomitant use of benzodiazepines.

Another significant finding of our study is the trend of opioid use over period of time. In the recently published USRDS analysis, Kimmel et al. indicated a much greater use of opioid (60% vs. 52% of population) compared to our study. However, this discrepancy in the findings is better explained when examining the data with reference to the similar trends in general population, especially a large dip seen in 2011 [40], attributed to increased awareness of dangers of prescription opioids, introduction of prescription drug monitoring program, and pain clinic regulations. Unfortunately, despite this decrease, we show that the number of hospitalizations in ESRD patients due to opioid overdose continued to rise throughout our study period, likely reflecting a significantly higher burden of comorbidities controlling the outcomes among ESRD patients.

The biggest aspect of our study is the analysis of safety for the concomitant use of benzodiazepines. While few studies have looked at the use of benzodiazepines in ESRD, no prior study has examined the safety of their concomitant use with opioids [41]. A study from the Office of Surveillance and Epidemiology, US FDA, in 2016 [42] demonstrated that the concomitant use of benzodiazepines and opioids in 2014 was around 9.6% in general population, but these rates were 30% in ESRD population in our study. A plausible explanation for this could be the higher prevalence of anxiety and other mental health issues in ESRD patients compared to the general population necessitating the legitimate need for benzodiazepines prescriptions. Our study also shows that the concomitant use of both drugs is associated with a 2.4-fold higher risk of hospitalization with opioid overdose. Simultaneous use of opioids and benzodiazepines has been shown to increase, in a dose-dependent fashion, respiratory depression and risk of death from drug overdose [43]. In 2016, the FDA issued a black box warning for concomitant use of the two classes of drugs [44]. It remains to be seen if this black box warning makes a significant difference in the concomitant prescription of both classes of drugs in the ESRD population.

We also looked at the hospitalization events and analyzed the individual opioids prescribed within 30, 60, or 90 days prior to hospitalization. We found that all opioids were associated with higher odds of hospitalization due to opioid overdose. In addition, the safety profile of the opioid for use in the ESRD population had little relationship with the risk of hospitalization due to an opioid overdose. The opioid with the lowest odds ratio for hospitalization was hydrocodone followed closely by hydromorphone. These two opioids comprised more than half of all opioid prescriptions and are typically classified as “use with caution” and “safe to use,” respectively. Opioids known to be unsafe for use in ESRD patients and corroborated by individual risk association in this study (such as morphine and meperidine) continue to be prescribed (comprising 2.5% of prescriptions in 2012).

It was surprising to see that methadone was associated with very high odds of hospitalization, despite being considered a safe drug based on pharmacokinetics. Methadone is a difficult analgesic to manage and needs certain skill sets from the prescribers with appropriate follow-ups to ensure safety of patients. Significant inter- and intra-individual variability in methadone metabolism could explain the discrepancy [45] with the hospitalization rate. Methadone is also used as part of substance abuse program, and some of these cases of overdose could be on that program, but this data is not readily available and difficult to capture and match with USRDS dataset.

Opioids to be used with caution, such as oxycodone and oxymorphone, were associated with OR > 3 of hospitalization. Oxycodone was the second most commonly prescribed opioid after hydrocodone. Overall, 40.4% of all opioid prescriptions were written for medications, which were associated with at least a moderate risk (OR of 2 and above) for hospitalization with opioid overdose. In essence, there is no “safe opioid” for use in hemodialysis patients, and the nomenclature is misleading. In fact, the WHO analgesic ladder modified for ESRD recommends methadone, fentanyl, oxycodone, and hydromorphone as step 3 for treatment of severe pain [8]. There are always legitimate reasons for opioid prescriptions, and all opioid prescriptions should not be viewed in the context of misuse. A societal and cultural acceptance of opioids plays a role in the acceptance of opioids for moderate-to-severe pain by both prescribers and the patients alike. The same goes for benzodiazepines too. This highlights the need for education of opioid prescribers regarding safety and selection of individual opioids in ESRD population. There is widespread prevalence of chronic pain, which is not adequately treated; in ESRD patients, opioids should not be withheld for uncontrolled pain for fear of complications. Interestingly, propoxyphene was found to be associated with a lower risk of hospitalization with opioid overdose; however, the association was not significant. It was banned by FDA in 2010 due to significant cardiac toxicity [46].

We did not look at prescriber profile or their education, background or competency in this study, but a 2015 study examined the prescription pattern of opioids by specialty. For similar time period (2007–2012) [47], it was found that primary care physicians wrote 11.2% and nonphysician providers wrote 44.5% of prescriptions. Hence, education regarding safety of these opioids in the ESRD population, targeting these providers, is paramount. Another limitation of our study is that we included patients only on Medicare (A and B) and part D. It is possible that patients covered by other insurance received opioid prescription not captured by our study.However, considering that the majority of hemodialysis patients have Medicare coverage, this number should be small, and possibly, would lead to an underestimation of opioid use.

In the current climate of opioid crisis, nonopioid options should always be considered first. Physical therapy and massage therapy may help with improving physical pain and range of motion. Psychotherapy including cognitive behavioral therapy may help with coping skills. There is a plethora of interventional pain procedures for which a referral to an interventional pain medicine expert could be valuable. Acetaminophen and NSAIDs are safe to use with dose adjustment and monitoring for gastrointestinal side effects [48, 49]. Adjuvants such as antiepileptics, antidepressants, and topical anesthetics can be considered.

This study demonstrates that the concept of safe, use with caution, and unsafe opioids in ESRD, while meaningful with regard to pharmacokinetics, is less meaningful with regard to resultant hospitalizations for opioid overdose. Concomitant opioid and benzodiazepine prescriptions even for legitimate reasons should be strictly monitored, and continuous prescriber education as well as competency improvement and assessment may play an important role in ensuring patient safety. More well-planned prospective studies involving multiple stake holders, including palliative care experts, will help to validate the findings of our retrospective study as well as suggest a better approach for managing opioids and benzodiazepines in ESRD patients.

## Figures and Tables

**Table 1:** Demographic and clinical characteristics of study cohort.

Characteristics	All patients	Patients without opioid prescription	Patients with at least one opioid prescription	*p* value	
	*n* = 643,859	*n* = 163,399	*n* = 480,460		*n* missing
Age, mean ± SD	62 ± 15	65 ± 15	61 ± 15	<0.001	

Gender, female *n* (%)	302,489 (47)	67,758 (42)	234,731 (49)	<0.001	74

Race, black, *n* (%)	223,264 (35)	52,997 (32)	170,267 (35)	<0.001	

BMI, mean ± SD	29 ± 8	28 ± 8	29 ± 8	<0.001	

Primary cause of ESRD, *n* (%)					
Diabetes	304,753 (47)	75,446 (46)	229,307 (48)	<0.001	
Hypertension	185,800 (29)	49,757 (30)	136,043 (28)		
Glomerulonephritis	63,178 (10)	14,381 (9)	48,797 (10)		
Cystic kidney	12,592 (2)	3,068 (2)	9,524 (2)		
Urologic disorders	8,543 (1)	2,219 (1)	6,324 (1)		
Others	45,403 (7)	11,522 (7)	33,881 (7)		

Comorbidity, *n* (%)					
Hypertension	543410 (86.2)	136,963 (85.6)	406,447 (86.4)	<0.001	13,267
Diabetes	351,024 (54.9)	86,932 (53.8)	264,092 (55.3)	<0.001	4,474
Atherosclerotic heart disease	127,296 (19.9)	34,025 (21.0)	93,271 (19.5)	<0.001	4,488
Chronic heart failure	197,713 (31.4)	50,826 (31.8)	146,887 (31.2)	<0.001	13,276
Cerebrovascular disease	58,006 (9.2)	15,500 (9.7)	42,506 (9.0)	<0.001	13,267
Peripheral vascular disease	82,008 (13.0)	20,671 (12.9)	61,337 (13.0)	0.261	13,277
Drug dependence	8,540 (1.4)	1,442 (0.9)	7,098 (1.5)	<0.001	13,277
Alcohol dependence	9,213 (1.5)	2,186 (1.4)	7,027 (1.5)	<0.001	13,277
Smoking	40,384 (6.4)	7,692 (4.8)	32,692 (6.9)	<0.001	13,277

Medication use, *n* (%)					
Benzodiazepine	162,713 (25.3)	18,529 (11.3)	144,184 (30.0)	<0.001	

**Table 2:** Association between clinical characteristics and having at least one opioid.

	Referent	Unadjusted		Multivariable logistic regression	
		OR (95% CI)	*p* value	OR (95% CI)	*p* value
Age		0.983 (0.983–0.984)	<0.001	0.984 (0.983–0.984)	<0.001

Female	Male	1.352 (1.336–1.367)	<0.001	1.419 (1.402–1.436)	<0.001

Race					
African Americans	White	1.093 (1.080–1.107)	<0.001	1.030 (1.017–1.044)	<0.001
Asian		0.602 (0.587–0.618)		0.626 (0.610–0.643)	
Native Americans		0.969 (0.919–1.020)		0.918 (0.870–0.969)	
Others		0.529 (0.485–0.578)		0.494 (0.452–0.541)	
Unknown		0.537 (0.302–0.954)		0.402 (0.220–0.734)	

Primary disease ESRD					
Diabetes	Other causes	1.070 (1.058–1.083)	<0.001	1.037 (1.021–1.054)	<0.001
Cystic kidney		1.089 (1.044–1.136)		0.990 (0.948–1.035)	
Urologic disorders		1.004 (0.955–1.055)		1.079 (1.025–1.136)	
BMI		1.021 (1.021–1.022)	<0.001		
Hypertension		1.063 (1.046–1.080)	<0.001	1.052 (1.035–1.070)	<0.001
Diabetes		1.074 (1.062–1.086)	<0.001	1.074 (1.057–1.092)	<0.001
Atherosclerotic heart disease		0.915 (0.902–0.928)	<0.001		
Chronic heart failure		0.974 (0.962–0.986)	<0.001	1.032 (1.018–1.045)	<0.001
Cerebrovascular disease		0.925 (0.907–0.943)	<0.001	0.972 (0.952–0.992)	0.005
Peripheral vascular disease		1.010 (0.993–1.027)	0.261	1.064 (1.045–1.083)	<0.001
Drug dependence		1.681 (1.588–1.780)	<0.001	1.309 (1.232–1.390)	<0.001
Alcohol dependence		1.094 (1.042–1.148)	<0.001	0.940 (0.893–0.990)	0.019
Smoking		1.477 (1.440–1.516)	<0.001	1.321 (1.286–1.357)	<0.001
Benzodiazepine		3.334 (3.279–3.390)	<0.001	3.271 (3.216–3.326)	<0.001

**Table 3:** Frequencies of adverse events according to opioid prescription use.

	All *N* (%)	Patients without opioid prescription *N* (%)	Patients with at least one opioid prescription *N* (%)	*p* value
	(*n* = 643,859)	(*n* = 163,399)	(*n* = 480,460)	
Patients with at least one hospital admission with a diagnosis of opioid overdose, *n* (%)	3,370 (0.52)	139 (0.09)	3,231 (0.67)	<0.001
Death due to drug overdose, *n* (%)	155 (0.02)	18 (0.01)	137 (0.03)	<0.001
Death due to opioid overdose (definition: death due to drug overdose during hospitalization with a diagnosis of opioid overdose), *n* (%)	6 (0.00)	1 (0.00)	5 (0.00)	0.624
Total LOS, median (IQR)	28 (12–59)	22 (9–48)	31 (13–62)	<0.001
Total ICU LOS, median (IQR)	6 (2–16)	5 (1–14)	6 (2–16)	<0.001
LOS in patients with opioid overdose, median (IQR)	4 (2–8)	5 (3–10)	4 (2–8)	0.326
ICU LOS in patients with opioid overdose, median (IQR)	2 (1–4)	2 (1–5)	2 (1–4)	0.144
Annual Medicare cost per patient ($1000), median (IQR)	43 (23–70)	33 (14–57)	46 (26–73)	<0.001

IQR, interquartile range; ICU, intensive care unit; LOS, length of stay.

**Table 4:** Proposed classification of opioids based on risk for hospitalization due to opioid overdose in ESRD patients on hemodialysis.

Original classification	Drug	Hospitalization within 30 days	Hospitalization within 60 days	Hospitalization within 90 days	Proposed classification^^^
		OR (95% CI)	OR (95% CI)	OR (95% CI)	
Safe	Hydromorphone	2.5 (2.2–2.8)	1.9 (1.7–2.1)	1.7 (1.5–1.9)	Moderate risk
	Fentanyl	3.0 (2.7–3.6)	2.8 (2.5–3.1)	2.7 (2.4–3.0)	High risk
	Methadone	6.0 (5.2–6.9)	5.3 (4.7–6.1)	4.8 (4.2–5.4)	Very high risk

Caution	Buprenorphine	5.4 (2.7–10.5)	3.9 (2.1–7.2)	4.3 (2.5–7.2)	Very high risk
	Hydrocodone	1.9 (1.8–2.0)	1.7 (1.6–1.8)	1.7 (1.6–1.8)	Low risk
	Oxycodone	3.1 (2.9–3.3)	2.7 (2.6–2.9)	2.6 (2.4–2.7)	High risk
	Oxymorphone	4.6 (3.0–7.1)	4.3 (2.9–6.2)	3.4 (2.4–4.9)	Very high risk

Unsafe	Meperidine	3.5 (1.7–7.1)	2.6 (1.4–4.9)	2.6 (1.5–4.6)	High risk
	Morphine	6.9 (6.2–7.6)	5.8 (5.2–6.4)	5.2 (4.7–5.7)	Very high risk
	Codeine	2.0 (0.8–4.8)	2.0 (1.0–4.3)	1.8 (0.9–3.6)	N/A^*∗*^
	Tapentadol	1.8 (0.2–12.8)	1.7 (0.4–6.9)	2.0 (0.6–6.3)	N/A^*∗∗*^
	Propoxyphene	0.8 (0.6–1.0)	0.8 (0.6–0.9)	0.7 (0.6–0.9)	N/A^*∗∗∗*^
	Benzodiazepine	1.5 (1.4–1.7)	1.5 (1.4–1.7)	1.5 (1.4–1.6)	

OR, odds ratio of hospitalization due to opioid overdose; CI, confidence interval. Multivariable logistic regression models included all opioids and benzodiazepines. ^^^Based on risk stratification using OR at 30 days: low risk, OR 1–1.9; moderate risk, OR 2–2.9; high risk, OR 3–3.9; very high risk, OR 4 and above. ^*∗*^Not statistically significant, ^*∗∗*^not statistically significant and wide CI due to sparse data, and ^*∗∗∗*^drug no longer available in market.

## Data Availability

The USRDS Standard Analysis Files (SAFs) data used to support the findings of this study may be released upon application to the United States Renal Data System (USRDS), who can be contacted at https://www.usrds.org/request.aspx or by e-mail to USRDS@usrds.org.

## Supplementary Materials

Supplementary Materials Table 1S: Updated Classification of opioids for use in ESRD. Table 2S: frequency of opioid prescriptions from 2006 to 2012 according to generic name.
