# Correction to “Opioid Safety and Concomitant Benzodiazepine Use in End‐Stage Renal Disease Patients”

**DOI:** 10.1155/prm/9852394

**Published:** 2026-10-27

R. Ruchi, S. Bozorgmehri, T. Ozrazgat‐Baslanti, et al., “Opioid Safety and Concomitant Benzodiazepine Use in End‐Stage Renal Disease Patients,” *Pain Research and Management*, vol. 2019 (2019), https://doi.org/10.1155/2019/3865924.

In the article titled “Opioid Safety and Concomitant Benzodiazepine Use in End‐Stage Renal Disease Patients,” a reference was omitted in error.

More specifically, the USRDS core dataset used in the study should be included as reference [12]. The full citation should be

[“[12]. USRDS. 2014 USRDS Annual Data Report: Epidemiology of Kidney Disease in the United States. National Institutes of Health, National Institute of Diabetes and Digestive and Kidney Diseases, Bethesda, Maryland, 2014.”]

Therefore, the corresponding sentence in Section 2.1. should be corrected as follows:

[“We conducted a retrospective study of U.S. Renal Data System(USRDS) database (2014) using the standard analysis files.”

should read

“We conducted a retrospective study of U.S. Renal Data System(USRDS) database (2014) using the standard analysis files. [12]”]

In addition, the *Disclosure* section should be modified as follows:

[“The interpretation and reporting of these data are the responsibility of the authors and in no way should be seen as an official policy or interpretation of the U.S. government.”

should read

“The data reported here have been supplied by the United States Renal Data System (USRDS). The interpretation and reporting of these data are the responsibility of the author(s) and in no way should be seen as an official policy or interpretation of the U.S. government.”

We apologise for these errors.

