# Supplementary material for: Opioid Safety and Concomitant Benzodiazepine Use in End-Stage Renal Disease Patients
Source: Pain Res Manag. 2019 Oct 20;2019:3865924. doi: 10.1155/2019/3865924 (PMC6854236; doi:10.1155/2019/3865924)
Supplement: Supplementary Materials — Table 1S: Updated Classification of opioids for use in ESRD. Table 2S: frequency of opioid prescriptions from 2006 to 2012 according to generic name. [file 3865924.f1.docx]

***Supplement***

**Opioid classification based on pharmacokinetic profile**

We performed an extensive review of the literature to classify opioids based on their safety profile in patients with ESRD on chronic hemodialysis. Accordingly opioids were classified as follows:

**Opioids considered safe to use in patients on HD**

Hydromorphone is generally considered safe to use in ESRD patients. Davison et al described the pharmacokinetics of hydromorphone in 12 anuric patients on HD, and found that the drug itself did not substantially accumulate during interdialytic period. Its metabolite hydromorphone 3 glucuronide did, but was effectively removed during dialysis. Overall, no significant opioid toxicity was noted and hydromorphone was deemed safe and effective for HD patients^12,13^.

Fentanyl: fentanyl has a large volume of distribution and is not dialyzable. It also has a short plasma half-life. 99% of fentanyl is metabolized in the liver to norfentanyl, which is an inactive metabolite. Hence, fentanyl appears to be safe to use in patients with kidney disease. Its safety has been demonstrated both via infusion and transdermal route in HD patients^14,15^.

Methadone has also been considered safe in patients on hemodialysis, based on its pharmacokinetic profile^13,16^. Almost all of the drug is excreted in the feces in anuric patients on hemodialysis without change in plasma levels^17^.

**Opioids considered unsafe to use in patients on HD**

Morphine is metabolized primarily in the liver to morphine 3 glucuronide (M3G) and morphine 6 glucuronide (M6G)^18^, which are renally excreted and hence accumulate in renal failure. M6G is an active metabolite that crosses the blood brain barrier and diffuses out slowly resulting in prolonged CNS depressive effects, and accounting for its poor clearance on dialysis^19-21^. Administration of morphine in ESRD is associated with respiratory depression requiring mechanical ventilation and altered mental status, and thus is best avoided.

Half life of codeine and its metabolites is significantly increased in ESRD patients on hemodialysis^22^, corroborated by reports of severe respiratory failure in patients on dialysis, and hence codeine is generally considered unsafe in ESRD patients^23^.

Meperidine (pethidine or Demerol), is converted to an active metabolite normeperidine which is known to accumulate in patients with renal failure and cause CNS excitation and seizures. Similarly, norpropoxyphene, an active metabolite of propoxyphene accumulates in patients with kidney disease and cause CNS toxicity. Both are therefore considered unsafe in ESRD and are best avoided^24-28^.

Tapentadol (nucynta) is a newer opioid, with no active metabolite. However, it has not been studied in patients on dialysis or those with GFR <30, and is currently not recommended in these patients. It is known to cause vasodilation and resultant hypotension in cumulative doses. Some nephrotoxicity has also been reported in animal models^29,30^.

**Opioids to be used with caution**

Hydrocodone is semi-synthetic congener of codeine. It is widely used in US in combination with acetaminophen, and is metabolized to active metabolites that can accumulate in renal failure. While use is not recommended in patients with ESRD who are not on dialysis, no data is available on its safety and clearance in patients who are on hemodialysis. The drug insert itself recommends using in lower doses^31^.

Oxycodone, also widely used alone or in combination with acetaminophen, has a high volume of distribution and is 50% protein bound. A study looked at levels of oxycodone and its metabolites oxymorphone and noroxycodone 2 hrs into hemodialysis in single patient, and found that they were all dialyzable to some extent. However, rebound and post dialysis levels were not studied. In a separate study, Samolksy Dekel BG et al reported the clearance of oxycodone in 20 patients and found only 22% and 17% reduction in arterial levels of oxycodone and noroxycodone respectively, after hemodialysis. Overall data for use of oxycodone in HD patients is limited^32,33^.

Buprenorphine (marketed as subutex or butrans patch, also available in combination with naloxone as suboxone)

In patients with renal failure, buprenorphine concentration was comparable to those without renal failure, however the concentration of norbuprenorphine and buprenorphine 3 glucouronide were increased by 4 and 15 times respectively^34^.

In contrast, Filitz et al reported levels of buprenorphine and norbuprenorhine that were unchanged after dialysis. However they used a transdermal formulation and norbuprenorphine levels were actually undetectable before dialysis in 7/10 patients^35^.

Data on nalbuphine is also limited, but one study showed elevated levels in ESRD patients, with minimal clearance on dialysis^36^.

**References:**

1. Davison SN. Pain in hemodialysis patients: prevalence, cause, severity, and management. *American journal of kidney diseases : the official journal of the National Kidney Foundation.* 2003;42(6):1239-1247.

2. Brown EA, Gower PE. Joint problems in patients on maintenance hemodialysis. *Clinical nephrology.* 1982;18(5):247-250.

3. Weisbord SD, Fried LF, Arnold RM, et al. Prevalence, severity, and importance of physical and emotional symptoms in chronic hemodialysis patients. *Journal of the American Society of Nephrology : JASN.* 2005;16(8):2487-2494.

4. Fainsinger RL, Davison SN, Brenneis C. A supportive care model for dialysis patients. *Palliative medicine.* 2003;17(1):81-82.

5. Davison SN, Jhangri GS. The impact of chronic pain on depression, sleep, and the desire to withdraw from dialysis in hemodialysis patients. *Journal of pain and symptom management.* 2005;30(5):465-473.

6. Tohme F, Mor MK, Pena-Polanco J, et al. Predictors and outcomes of non-adherence in patients receiving maintenance hemodialysis. *International urology and nephrology.* 2017.

7. McClellan WM, Anson C, Birkeli K, Tuttle E. Functional status and quality of life: predictors of early mortality among patients entering treatment for end stage renal disease. *Journal of clinical epidemiology.* 1991;44(1):83-89.

8. Barakzoy AS, Moss AH. Efficacy of the world health organization analgesic ladder to treat pain in end-stage renal disease. *Journal of the American Society of Nephrology : JASN.* 2006;17(11):3198-3203.

9. Bailie GR, Mason NA, Bragg-Gresham JL, Gillespie BW, Young EW. Analgesic prescription patterns among hemodialysis patients in the DOPPS: potential for underprescription. *Kidney international.* 2004;65(6):2419-2425.

10. O'Connor NR, Corcoran AM. End-stage renal disease: symptom management and advance care planning. *American family physician.* 2012;85(7):705-710.

11. Dean M. Opioids in renal failure and dialysis patients. *Journal of pain and symptom management.* 2004;28(5):497-504.

12. Davison SN, Mayo PR. Pain management in chronic kidney disease: the pharmacokinetics and pharmacodynamics of hydromorphone and hydromorphone-3-glucuronide in hemodialysis patients. *Journal of opioid management.* 2008;4(6):335-336, 339-344.

13. Perlman R, Giladi H, Brecht K, et al. Intradialytic clearance of opioids: methadone versus hydromorphone. *Pain.* 2013;154(12):2794-2800.

14. Mercadante S, Caligara M, Sapio M, Serretta R, Lodi F. Subcutaneous fentanyl infusion in a patient with bowel obstruction and renal failure. *Journal of pain and symptom management.* 1997;13(4):241-244.

15. Han J, Saraf SL, Gordeuk VR, Gowhari M. Safety of chronic transdermal fentanyl use in patients receiving hemodialysis. *American journal of health-system pharmacy : AJHP : official journal of the American Society of Health-System Pharmacists.* 2016;73(13):947-948.

16. Opdal MS, Arnesen M, Muller LD, et al. Effects of Hemodialysis on Methadone Pharmacokinetics and QTc. *Clinical therapeutics.* 2015;37(7):1594-1599.

17. Kreek MJ, Schecter AJ, Gutjahr CL, Hecht M. Methadone use in patients with chronic renal disease. *Drug and alcohol dependence.* 1980;5(3):197-205.

18. Osborne R, Joel S, Grebenik K, Trew D, Slevin M. The pharmacokinetics of morphine and morphine glucuronides in kidney failure. *Clinical pharmacology and therapeutics.* 1993;54(2):158-167.

19. Osborne RJ, Joel SP, Slevin ML. Morphine intoxication in renal failure: the role of morphine-6-glucuronide. *British medical journal (Clinical research ed.).* 1986;292(6535):1548-1549.

20. Bodd E, Jacobsen D, Lund E, Ripel A, Morland J, Wiik-Larsen E. Morphine-6-glucuronide might mediate the prolonged opioid effect of morphine in acute renal failure. *Human & experimental toxicology.* 1990;9(5):317-321.

21. Angst MS, Buhrer M, Lotsch J. Insidious intoxication after morphine treatment in renal failure: delayed onset of morphine-6-glucuronide action. *Anesthesiology.* 2000;92(5):1473-1476.

22. Guay DR, Awni WM, Findlay JW, et al. Pharmacokinetics and pharmacodynamics of codeine in end-stage renal disease. *Clinical pharmacology and therapeutics.* 1988;43(1):63-71.

23. Talbott GA, Lynn AM, Levy FH, Zelikovic I. Respiratory arrest precipitated by codeine in a child with chronic renal failure. *Clinical pediatrics.* 1997;36(3):171-173.

24. Chan GL, Matzke GR. Effects of renal insufficiency on the pharmacokinetics and pharmacodynamics of opioid analgesics. *Drug intelligence & clinical pharmacy.* 1987;21(10):773-783.

25. Szeto HH, Inturrisi CE, Houde R, Saal S, Cheigh J, Reidenberg MM. Accumulation of normeperidine, an active metabolite of meperidine, in patients with renal failure of cancer. *Annals of internal medicine.* 1977;86(6):738-741.

26. Armstrong PJ, Bersten A. Normeperidine toxicity. *Anesthesia and analgesia.* 1986;65(5):536-538.

27. Hsu CH, Lin TC, Lu CC, Lin SH, Ho ST. Clearance of meperidine and its metabolite normeperidine in hemodialysis patients with chronic noncancer pain. *Journal of pain and symptom management.* 2014;47(4):801-805.

28. Nickander RC, Emmerson JL, Hynes MD, Steinberg MI, Sullivan HR. Pharmacologic and toxic effects in animals of dextropropoxyphene and its major metabolite norpropoxyphene: a review. *Human toxicology.* 1984;3 Suppl:13s-36s.

29. Barbosa J, Faria J, Leal S, et al. Acute administration of tramadol and tapentadol at effective analgesic and maximum tolerated doses causes hepato- and nephrotoxic effects in Wistar rats. *Toxicology.* 2017;389:118-129.

30. Vadivelu N, Huang Y, Mirante B, et al. Patient considerations in the use of tapentadol for moderate to severe pain. *Drug, healthcare and patient safety.* 2013;5:151-159.

31. Murtagh FE, Chai MO, Donohoe P, Edmonds PM, Higginson IJ. The use of opioid analgesia in end-stage renal disease patients managed without dialysis: recommendations for practice. *Journal of pain & palliative care pharmacotherapy.* 2007;21(2):5-16.

32. Lee MA, Leng ME, Cooper RM. Measurements of plasma oxycodone, noroxycodone and oxymorphone levels in a patient with bilateral nephrectomy who is undergoing haemodialysis. *Palliative medicine.* 2005;19(3):259-260.

33. Samolsky Dekel BG, Donati G, Vasarri A, et al. Dialyzability of Oxycodone and Its Metabolites in Chronic Noncancer Pain Patients with End-Stage Renal Disease. *Pain practice : the official journal of World Institute of Pain.* 2017;17(5):604-615.

34. Hand CW, Sear JW, Uppington J, Ball MJ, McQuay HJ, Moore RA. Buprenorphine disposition in patients with renal impairment: single and continuous dosing, with special reference to metabolites. *British journal of anaesthesia.* 1990;64(3):276-282.

35. Filitz J, Griessinger N, Sittl R, Likar R, Schuttler J, Koppert W. Effects of intermittent hemodialysis on buprenorphine and norbuprenorphine plasma concentrations in chronic pain patients treated with transdermal buprenorphine. *European journal of pain (London, England).* 2006;10(8):743-748.

36. Hawi A, Alcorn H, Jr., Berg J, Hines C, Hait H, Sciascia T. Pharmacokinetics of nalbuphine hydrochloride extended release tablets in hemodialysis patients with exploratory effect on pruritus. *BMC nephrology.* 2015;16:47.

37. Kimmel PL, Fwu CW, Abbott KC, Eggers AW, Kline PP, Eggers PW. Opioid Prescription, Morbidity, and Mortality in United States Dialysis Patients. *Journal of the American Society of Nephrology : JASN.* 2017;28(12):3658-3670.

38. Centers for Disease Control and Prevention. Annual Surveillance Report of Drug-Related Risks and Outcomes. 2017; https://www.cdc.gov/drugoverdose/pdf/pubs/2017-cdc-drug-surveillance-report.pdf.

39. Olivo RE, Hensley RL, Lewis JB, Saha S. Opioid Use in Hemodialysis Patients. *American journal of kidney diseases : the official journal of the National Kidney Foundation.* 2015;66(6):1103-1105.

40. Guy GP, Jr., Zhang K, Bohm MK, et al. Vital Signs: Changes in Opioid Prescribing in the United States, 2006-2015. *MMWR. Morbidity and mortality weekly report.* 2017;66(26):697-704.

41. Wyne A, Rai R, Cuerden M, Clark WF, Suri RS. Opioid and benzodiazepine use in end-stage renal disease: a systematic review. *Clinical journal of the American Society of Nephrology : CJASN.* 2011;6(2):326-333.

42. Hwang CS, Kang EM, Kornegay CJ, Staffa JA, Jones CM, McAninch JK. Trends in the Concomitant Prescribing of Opioids and Benzodiazepines, 2002-2014. *American journal of preventive medicine.* 2016;51(2):151-160.

43. Park TW, Saitz R, Ganoczy D, Ilgen MA, Bohnert AS. Benzodiazepine prescribing patterns and deaths from drug overdose among US veterans receiving opioid analgesics: case-cohort study. *BMJ (Clinical research ed.).* 2015;350:h2698.

44. U.S. Food & Drug Administration. FDA News Release. 2016; https://www.fda.gov/NewsEvents/Newsroom/PressAnnouncements/ucm518697.htm.

45. Gadel S, Friedel C, Kharasch ED. Differences in Methadone Metabolism by CYP2B6 Variants. *Drug metabolism and disposition: the biological fate of chemicals.* 2015;43(7):994-1001.

46. Administration USFD. FDA Drug Safety Communication: FDA recommends against the continued use of propoxyphene. 2010; https://www.fda.gov/Drugs/DrugSafety/ucm234338.htm. Accessed 4.5.2018.

47. Levy B, Paulozzi L, Mack KA, Jones CM. Trends in Opioid Analgesic-Prescribing Rates by Specialty, U.S., 2007-2012. *American journal of preventive medicine.* 2015;49(3):409-413.

48. Prescott LF, Speirs GC, Critchley JA, Temple RM, Winney RJ. Paracetamol disposition and metabolite kinetics in patients with chronic renal failure. *European journal of clinical pharmacology.* 1989;36(3):291-297.

49. Cohen LM, Moss AH, Weisbord SD, Germain MJ. Renal palliative care. *Journal of palliative medicine.* 2006;9(4):977-992.

| **Table 1S. Updated Classification of opioids for use in ESRD*** | | |
| --- | --- | --- |
| Safe | Caution | Unsafe |
| FENTANYL | BUPRENORPHINE | CODEINE |
| HYDROMORPHONE | DIHYDROCODEINE | MEPERIDINE |
| METHADONE | HYDROCODONE | MORPHINE |
|  | OXYCODONE | PROPOXYPHENE |
|  | OXYMORPHONE | TAPENTADOL |

* Updated from the original AAFP classification^10^

| **Table 2S. Frequency of opioid prescriptions from 2006 to 2012 according to generic name** | | |
| --- | --- | --- |
| Generic Name | Frequency | Percent |
|  |  |  |
| Hydrocodone | 3,668,193 | 55.03 |
| Oxycodone | 1,753,959 | 26.31 |
| Propoxyphene | 484,881 | 7.27 |
| Fentanyl | 284,433 | 4.27 |
| Hydromorphone | 180,264 | 2.7 |
| Morphine | 161,328 | 2.42 |
| Methadone | 102,925 | 1.54 |
| Oxymorphone | 11,576 | 0.17 |
| Codeine | 7,436 | 0.11 |
| Buprenorphine | 5,880 | 0.09 |
| Meperidine | 3,966 | 0.06 |
| Tapentadol | 1,065 | 0.02 |
| Total | 6,665,906 |  |
